# Plague Reappearance in Algeria after 50 Years, 2003

**DOI:** 10.3201/eid1310.070284

**Published:** 2007-10

**Authors:** Eric Bertherat, Souad Bekhoucha, Saada Chougrani, Fathia Razik, Jean B. Duchemin, Leila Houti, Larbi Deharib, Corinne Fayolle, Banaouda Makrerougrass, Radia Dali-Yahia, Ramdan Bellal, Leila Belhabri, Amina Chaieb, Evgueni Tikhomirov, Elisabeth Carniel

**Affiliations:** * World Health Organization, Geneva, Switzerland; † University Hospital, Oran, Algeria; ‡ University of Medicine, Oran, Algeria; § Centre d'Etude et de Recherche pour les Meningocoques et les Schistosomiases, Niamey, Niger; ¶ Institut Pasteur, Paris, France; and #Institut Pasteur, Oran, Algeria

**Keywords:** Plague, Algeria, disease outbreaks, research

## Abstract

An outbreak of plague occurred in the region of Oran, Algeria, from June to July 2003. Algeria had not reported this disease for >50 years. Eighteen bubonic cases were identified, and *Yersinia pestis* was isolated from 6 patients. Except for the index case-patient, all patients recovered. Targeted chemoprophylaxis, sanitation, and vector control played a crucial role in controlling the outbreak. Epidemiologic and biomolecular findings strongly suggested the existence of a local animal reservoir during this period, but its origin (resurgence or re-importation) could not be determined. This sudden and unexpected reemergence of plague, close to an important commercial seaport, is a textbook illustration of a public health event of international importance. It also demonstrates that the danger of plague reoccurrence is not limited to the currently indexed natural foci.

## Abstract 2

Plague is not limited to the currently indexed natural foci in Algeria.

Plague is primarily a bacterial zoonosis affecting rodents. It is caused by *Yersinia pestis* and is transmitted from animal to animal by fleas. Humans usually become infected through the bite of an infected rodent flea. Bubonic plague, a severe infectious disease which, in the absence of appropriate antimicrobial drug therapy, can evolve to a rapidly fatal septicemia or pneumonia, can develop. A pneumonia form, which enables direct transmission to contacts, can be responsible for highly lethal outbreaks.

Currently, plague natural foci persist in Asia, the Americas, and Africa (where most human cases occur) (*1*). Plague foci have previously existed in the northern part of Africa but gradually disappeared in the last century, for unknown reasons. Libya is the only north African country that has experienced human cases in the past 40 years (*2*). In Algeria, archives report epidemics of plague as far back as the 14th century. These epidemics mainly affected ports, particularly that of Oran in 1556 and 1678 (3,000 deaths). In 1899, after an absence of nearly 100 years, plague reappeared in the port of Philippeville (now Skikda). Three large epidemics were subsequently reported in 1921 (185 cases), 1931 (76 cases), and 1944 (95 cases) as well as 158 sporadic cases. All but 2 cases occurred in ports (*3*,*4*). No natural focus of plague had ever been described in Algeria (*5*). We describe an outbreak of bubonic plague that occurred in 2003 in Algeria, where the last reported human case occurred in Oran in 1946 (*6*).

## Methods

During June 9–18, 2003, several patients with signs of severe infection and painful inflammatory adenopathy were admitted to the University Hospital of Oran. All came from Kehailia (35°29′N, 0°32′E), a village of 1,300 inhabitants 25 km south of Oran. After eliminating all other possible differential diagnoses, clinicians suspected plague. The diagnosis was confirmed on June 18 by results of analysis of a bubo (lymph node) aspirate. A technical crisis committee was set up, and a case definition was adopted (Table). Any patient with a febrile syndrome and adenopathy who resided in the prefecture of Oran was hospitalized.

**Table:** Plague case definition adopted by technical crisis committee, 2003 plague outbreak, Oran region, Algeria*

Case definition	Criteria
Suspected	Clinical and epidemiologic characteristics compatible with plague; or, observation of suspect microorganisms on direct examination of clinical samples
Probable	Suspected case with anti-F1 antibodies in patient’s blood; or, suspected case with a positive RDT without isolation of *Yersinia pestis* or in the absence of other cases reported in a radius of 10 km around the case
Confirmed	Culture positive for *Y. pestis*; or, RDT positive and *Y. pestis* isolated from patients living in a radius of 10 km around the case

*RDT, rapid diagnostic test.

Clinical samples collected from patients (blood, bubo aspirate, cerebrospinal fluid) were sent to the Microbiology Department, University Hospital, Oran. Several of the initial cases were first diagnosed with the rapid diagnostic test (RDT) for plague developed by the Institut Pasteur (*7*); however, all samples were also examined with standard bacteriologic methods. Direct examination of smears was performed after Wayson and Gram staining. Blood samples were cultured in Castaneda medium for at least 10 days at 28°C and examined daily. Suspected samples were inoculated into brain heart infusion and peptone broth and streaked on blood agar and cefsulodin-irgasan-novobiocin (Merck, Rahway, NJ, USA) plates. All media were incubated at 28°C. Bacterial identification was conducted with API 20 E strips (Analytab Products, Syosset, NY, USA) or individual tests in tubes. The biovar was determined (*8*). Antimicrobial drug susceptibility testing (ampicillin, amoxicillin-clavulanic acid, cefazolin, cefotaxime, gentamicin, amikacin, sulfamethoxazole, doxycycline) was conducted according to the technique of the Clinical and Laboratory Standards Institute (www.clsi.org). The serodiagnosis was determined by the ELISA-F1 technique (*9*). Serum samples from 30 study participants who had not contracted the disease but lived in the same area as the patients were used to determine the positive threshold of the technique. A serum was regarded as negative if its optical density at 490 nm (OD_490_) was lower than a threshold defined as the mean (M) OD_490_ value of normal sera + 3 standard deviations (SD): OD_490_ < M + 3 SD. Sera with OD higher than this threshold were regarded as weak when the ratio R = OD_490_/(M + 3SD) was <2 and positive if R was >2.

## Results

On June 9, 2003, a 19-year-old shepherd living in Kehailia was hospitalized with signs of septic shock (patient no. 2) (Appendix Table). He had been treated at home unsuccessfully with cephalosporins for inguinal adenopathy and fever during the previous 8 days. In the same village, 6 similar cases (nos. 3–8) occurred in the following days, until the diagnosis of plague was suspected and confirmed on June 18, first by RDT and then by isolation of a bacterium that had all the characteristics of *Y. pestis* biovar Orientalis and was susceptible to the antimicrobial agents tested. The epidemiologic investigation uncovered the index patient (no. 1), an 11-year-old child from Kehailia who was a cousin of case-patient 2. On June 2, an inguinal adenopathy with fever developed, and patient 2 was transferred to the hospital. He died 3 hours later, without a precise diagnosis.

Following the sanitation measures (reduction of rodent harborage, garbage removal, and vector control) implemented in Kehailia, no new cases of plague were reported in this locality after June 17. On June 19, a woman living in the suburbs of Oran (Hai Oussama) was hospitalized with bubonic plague (patient 9). The investigation showed that she had gone to Kehailia in the preceding days to consult a healer. Five cases of bubonic plague (nos. 10, 11, 14, 15, and 17) subsequently occurred from June 21 to July 16 among persons living in villages around Kehailia.

On June 28, a farmer and his wife (patients 12 and 13) who resided in Ain Temouchent, 50 km west of Kehailia (Figure), were hospitalized in Oran for symptoms suggestive of plague. The patients reported that they had not left their farm during the weeks preceding their illness. On July 1, a child from Beni Saf, on the Mediterranean coast 100 km southwest of Kehailia (Figure), had clinical signs of bubonic plague and a positive RDT result (patient 16). Neither he, nor his parents, had gone to the area of Kehailia or Ain Temouchent during the previous days. The last case (patient 18) occurred on July 22. The patient, a hunter who lived in Oran, had walked in the forest of M’sila, 30 km northwest of Kehailia, a few days before onset of his clinical signs.

**Figure:**
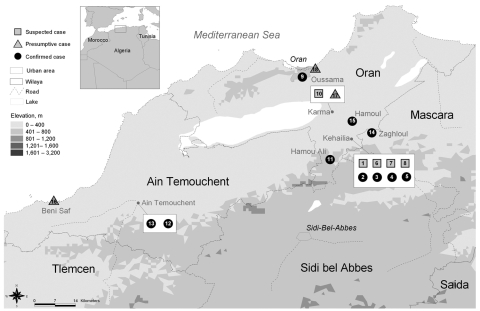
Geographic distribution of plague cases, Oran region, June–July 2003. Boundaries and names shown and the designations used on this map do not imply the expression of any opinion whatsoever on the part of the World Health Organization (WHO) concerning the legal status of any country, territory, city, or area or of its authorities, or concerning the delimitation of its frontiers or boundaries. Dotted lines on maps represent approximate border lines for which there may not yet be full agreement. Data source: Ministry of Health Algeria. Map production: Public Health Mapping and GIS, Communicable Diseases, WHO. Copyright WHO, 2006. Used with permission.

Altogether, 18 cases were identified June 4–July 22, 2003: 10 confirmed, 3 probable, and 5 suspected (or 12 confirmed, 2 probable, and 4 suspected, according to the new World Health Organization case definition [*1*]). Most of the patients lived in unsanitary conditions, in close contact with livestock, and in the vicinity of storage areas of grain and fodder. In Kehailia, all the case-patients resided in different dwellings located within a 200-m radius. None of them reported direct contact with rodents. Sixteen of the 18 patients had an inguinal bubo, indicative of a flea bite on the leg. A septicemic form of plague developed in patients 1 and 2. Patient 1 died very soon after hospital admission. Patient 2 was admitted with a severe fever and neurologic syndrome and fell into a deep coma, despite broad-spectrum antimicrobial drug treatment that included vancomycin, cefotaxime, and gentamicin. He recovered from the coma 48 hours after treatment with ciprofloxacin (500 mg 2×/d for 30 days) was completed (F. Razik et al., unpub. data). No case of secondary pulmonary dissemination was observed. Other plague patients were treated with either doxycycline for adults (200 mg/d for 10 days) or cotrimoxazole for children (40 mg/kg/d for 10 days). All recovered without sequelae.

On the whole, 60 bubo aspirates, 143 blood samples, 6 sputum samples, and 2 cerebrospinal fluid samples were analyzed. In 5 samples, smear stains suggested infection with *Y. pestis* (Appendix Table). Among the 18 patients, 12 had a positive RDT result, but *Y. pestis* was isolated from only 6 patients: 5 from bubo aspirates and 1 from the blood culture of a patient whose bubo was too small to be punctured (patient 13). Results of ELISA-F1 serologic test conducted on the serum samples from 15 of the 18 patients were strongly positive 3 times and slightly positive 3 times (Appendix Table).

## Discussion

Epidemiologic investigation did not identify any other plague patients before patient 1. It is unlikely that other cases occurred and remained undetected during this period since plague, even in its bubonic form, is a severe infection with high fatality rates.

For the first time, the RDT was used in an epidemic situation outside of Madagascar, where it was developed. The case definition had to take into account this particularity. The bacteriologic diagnosis is a long procedure (at least 4 days) and, in this epidemic context, RDT contributed to the effectiveness of the response. Of the 44 RDTs that were conducted, 12 had positive results; by contrast, culture was positive only for 6. Among the 15 patients for whom a serologic test was conducted (Appendix Table), a specific antibody response developed only in 6. This absence of specific antibodies can be explained by the fact that serum specimens were taken before the appearance of anti-F1 immunoglobulin G, or by a rapid administration of antimicrobial drugs, which stopped development of an immune response. The 3 clearly seropositive patients were those from whom a positive culture was obtained.

The outbreak occurred in a poor rural settlement, with inadequate sanitation. The residents observed an increase in the population of commensal rodents, which is often associated with the harvesting period, but no unusual rodent mortality was noted during the weeks preceding the outbreak. The appearance during the same week of 2 new cases in Ain Temouchent (50 km west of Kehailia) and then 1 case in Beni Saf (100 km southwest of Kehailia) could not be explained. Nonetheless, the fact that the *Y. pestis* strains isolated in Kehailia and Ain Temouchent had identical pulsotypes (V. Chenal-Francisque et al., unpub. data) argues for a single focus and not for independent foci that emerged simultaneously.

A crisis committee designed and supervised a control strategy based on standardized case management, prophylactic treatment and follow-up of contacts sharing the same dwelling as plague patients, and vector control. Environmental sanitation measures in Kehailia contributed to reduction in the occurrence of new cases in this village. Intra- and peridomestic spraying with permethrin was conducted. Deltamethrin was dusted on the tracks and around the burrows of rodents located in a radius of 10 km around the dwelling of the patients. Uncontrolled killing of rats was prohibited.

No natural focus of plague had ever been described in Algeria. Past cases were always regarded as imported through the ports. The reappearance of human cases in this area can be explained in 2 ways: a recent importation of infected animals or a sudden manifestation of a natural focus that had remained silent for decades. It is noteworthy that Kehailia, the epicenter of the outbreak, is in the vicinity of flour mills built 4 years before the outbreak. These mills are supplied regularly with cereals by trucks arriving from the port of Oran. A part of this traffic was still run by railway a year before the outbreak, and a marshalling yard was installed a few kilometers from Kehailia. In 1919, this mode of importation was responsible for the plague outbreak that occurred 75 km south of the port of Skikda (*10*). The hypothesis of recent importation of the plague bacillus in Kehailia is therefore tempting but is tempered by the fact that 1) the grain is primarily imported from Europe, which is not affected by plague, and from North America where natural foci exist but have very limited areas of overlap with those regions where cereal grains are grown, 2) no higher mortality rate in the murine population of the port was noted, 3) no human cases occurred in this sector of the city, and 4) a 3IS–restriction fragment length polymorphism (*11*) analysis grouped these strains in a cluster clearly distinct from the strains isolated from Africa and America (V. Chenal-Francisque et al., unpub. data).

The geographic concentration of the cases in 2 foci, both contiguous in the mountainous area of Tessala, suggested the existence of a natural focus in this area. Moreover, *Meriones* are present in Tessala, and these rodents are a well-known potential reservoir of *Y. pestis* (*12*). The outbreak occurred at harvest time, and it is possible that the abrupt reduction in the source of food pushed the wild rodents to approach houses in which grain was stored.

The current challenge in terms of public health is to determine if this animal reservoir has disappeared or if it is well established in the ecosystem. The capture of 3 seropositive small mammals (2 *Mus musculus* and 1 *Aleterix algerius*) in July 2004 (J.L. Soares et al., unpub. data) and the identification of several *Y. pestis* infected fleas in the same area (*13*) favor the second option.

Beyond the local problem, the proximity of a possible natural reservoir of plague to Oran, a large international commercial port, raises the possibility of the risk for an urban outbreak. At the time of the investigation, the sanitation in the city and port were poor and rodents proliferated. These urban rodents could come in contact with infected rodents from rural areas in the uncontrolled dumps at the periphery or through a dry riverbed that penetrates as far as the city center. Because of Oran’s population density and the commercial activities of its seaport, a plague outbreak would have international implications.

This outbreak is a textbook illustration of the unexpected and sudden reemergence of an infectious disease epidemic that is potentially highly lethal. It also demonstrates that the danger of a plague outbreak is not limited to the currently indexed natural foci.

## Supplementary Material

Appendix Table Characteristics, clinical manifestations, and laboratory results of plague patients in Algeria, June-July 2003*
