# Supplementary material for: Plague Reappearance in Algeria after 50 Years, 2003
Source: Emerg Infect Dis. 2007 Oct;13(10):1459–62. doi: 10.3201/eid1310.070284 (PMC2851531; doi:10.3201/eid1310.070284)
Supplement: Appendix Table — Characteristics, clinical manifestations, and laboratory results of plague patients in Algeria, June-July 2003* [file 07-0284_appT-s1.pdf]

Appendix Table. Characteristics, clinical manifestations, and laboratory results of plague patients in Algeria, June-July 2003*

Patient no.	Sex	Age, y	Place of residence	Occ.	Date of admission	Clinical manifestations	Evolution	Laboratory confirmation: Direct examination	Culture	RDT	Sero 1 (bleeding date)	Sero 2	Remark	Class.
1	M	11	Kehailia	Pupil	Jun 4	Septicemia	Died	–	–	–	–	–	Cousin of patient no. 2	S
2	M	19	Kehailia	Shepherd	Jun 9	Septicemia Inguinal bubo	Recovered	Neg	Neg	Pos	Neg (Jul 12)	–	Cousin of patient no. 1	C
3	M	70	Kehailia	Farmer	Jun 13	Inguinal bubo	Recovered	Neg	Neg	Pos	Weak (Jul 7)	–	Head of the village	C
4	F	27	Kehailia	None	Jun 14	Inguinal bubo	Recovered	Pos	Pos	Pos	Pos (July 12)	–		C
5	M	26	Kehailia	Shepherd	Jun 15	Inguinal bubo	Recovered	Neg	Neg	Pos	Neg (Jul 7)	–		C
6	M	20	Kehailia	Trader	Jun 16	Inguinal bubo	Recovered	–	–	–	Neg (Jul 7)	–		S
7	M	20	Kehailia	None	Jun 17	Inguinal bubo	Recovered	–	–	–	Neg (Jul 7)	–		S
8	F	54	Kehailia	None	Jun 17	Inguinal bubo	Recovered	–	–	–	–	–		S
9	F	25	Hai Oussama	None	Jun 19	Inguinal bubo	Recovered	Neg	Pos	Pos	Neg (Jul 7)	–	Stay in Kehailia	C
10	M	27	Karma Es-Senia	Farmer	Jun 21	Inguinal bubo	Recovered	Pos	Neg	Neg	Neg (Jul 7)	–	3 km from Kehailia	S
11	M	55	Hama Ali	Mason	Jun 27	Inguinal bubo	Recovered	Pos	Pos	Pos	Neg (Jul 8)	–	2 km from Kehailia	C
12	M	58	Ain Temouchent	Shepherd	Jun 28	Inguinal bubo	Recovered	Pos	Pos	Pos	Pos (Jul 21)	Pos	Husband of 13	C
13	F	55	Ain Temouchent	None	Jun 28	Inguinal bubo	Recovered	–	Pos	–	Weak (Jul 21)	Pos	Wife of 12	C
14	F	2	Zeghloul	None	Jun 30	Inguinal bubo	Recovered	Neg	Neg	Pos	–	–	10 km from Kehailia	C
15	F	3	Hamoul	None	Jun 30	Inguinal bubo	Recovered	Pos	Pos	Pos	Pos (Jul 8)	Pos (11/08/03)	4 km from Kehailia	C
16	M	7	Beni Saf	None	Jul 1	Inguinal bubo	Recovered	Neg	Neg	Pos	Neg (Jul 7)	–	25 km from Kehailia	P
17	F	24	El Karma	Civil servant	Jul 16	Axillary bubo	Recovered	Neg	Neg	Pos	Weak (Jul 20)	–	Stay in the house of 8	P
18	M	20	Oran	Hunter	Jul 22	Inguinal bubo	Recovered	Neg	Neg	Pos	Neg (Jul 23)	–		P

*Occ., occupation; RDT, rapid diagnostic test; Class., classification; –, not done; Neg, negative; Pos, positive; C, confirmed case; S, suspected case; P, presumptive case.
